# Sociodemographic and socioeconomic patterns of chronic non-communicable disease among the older adult population in Ghana

**DOI:** 10.3402/gha.v7.21292

**Published:** 2014-04-15

**Authors:** Nadia Minicuci, Richard B. Biritwum, George Mensah, Alfred E. Yawson, Nirmala Naidoo, Somnath Chatterji, Paul Kowal

**Affiliations:** 1 National Research Council, Neuroscience Institute, Padova, Italy; 2 Department of Community Health, University of Ghana, Accra, Ghana; 3 Multi-Country Studies Unit, World Health Organization, Geneva, Switzerland; 4 Research Centre for Gender, Health and Ageing, University of Newcastle, Newcastle, Australia

**Keywords:** SAGE, Ghana, aging, health behavior, non-communicable disease

## Abstract

**Background:**

In Ghana, the older adult population is projected to increase from 5.3% of the total population in 2015 to 8.9% by 2050. National and local governments will need information about non-communicable diseases (NCDs) in this population in order to allocate health system resources and respond to the health needs of older adults.

**Design:**

The 2007/08 Study on global AGEing and adult health (SAGE) Wave 1 in Ghana used face-to-face interviews in a nationally representative sample of persons aged 50-plus years. Individual respondents were asked about their overall health, diagnosis of 10 chronic non-communicable conditions, and common health risk factors. A number of anthropometric and health measurements were also taken in all respondents, including height, weight, waist and hip circumferences, and blood pressure (BP).

**Results:**

This paper includes 4,724 adults aged 50-plus years. The highest prevalence of self-reported chronic conditions was for hypertension [14.2% (95% CI 12.8–15.6)] and osteoarthritis [13.8%, (95% CI 11.7–15.9)]. The figure for hypertension reached 51.1% (95% CI 48.9–53.4) when based on BP measurement. The prevalence of current smokers was 8.1% (95% CI 7.0–9.2), while 2.0 (95% CI 1.5–2.5) were infrequent/frequent heavy drinkers, 67.9% (95% CI 65.2–70.5) consume insufficient fruits and vegetables, and 25.7% (95% CI 23.1–28.3) had a low level of physical activity. Almost 10% (95% CI 8.3–11.1) of adults were obese and 77.6% (95% CI 76.0–79.2) had a high-risk waist-to-hip ratio (WHR). Risks from tobacco and alcohol consumption continued into older age, while insufficient fruit and vegetable intake, low physical activity and obesity increased with increasing age. The patterns of risk factors varied by income quintile, with higher prevalence of obesity and low physical activity in wealthier respondents, and higher prevalence of insufficient fruit and vegetable intake and smoking in lower-income respondents. The multivariate analysis showed that only urban/rural residence and body mass index (BMI) were common determinates of both self-reported and measured hypertension, while all other determinants have differing patterns.

**Conclusions:**

The findings show a high burden of chronic diseases in the older Ghanaian population, as well as high rates of modifiable health risk factors. The government could consider targeting these health behaviors in conjunction with work to improve enrolment rates in the National Health Insurance Scheme.

Ghana is among a number of sub-Saharan African countries experiencing epidemiological transition where chronic diseases are on the increase, including among the older population. This coincides with population aging experienced across all major regions of the world, including Africa (1). In sub-Saharan Africa, as a percentage of the total population, the current population of adults aged 60 years and older is 4.9%, increasing to 7.6% by 2050 (1). Within sub-Saharan Africa, Ghana is older than the average with 5.3% of older adults in the total population currently, increasing to 8.9% in 2050.

Given the demographic trends globally, the significance of chronic diseases among the aging population is increasing, with clear implications for the quality of life of older adults. The 2010 Global Burden of Disease Study provides evidence of the changing trends in disease patterns in sub-Saharan Africa, with considerable decreases in Disability Adjusted Life Years (DALYs)/100,000 population overall but a large increase in the percentage of DALYs attributable to chronic conditions, particularly in the older age groups (2). In Ghana, communicable, maternal, perinatal, and nutritional conditions currently constitute about 53% of non-fatal disease burden, non-communicable diseases (NCDs) constituted 41% and injuries 6% (2). As Ghana strives towards continued socioeconomic development through improved education, health, infrastructure, and technology, the health system will also need to shift to respond to the evolving demographic and epidemiological realities.

The adult population aged 60 years and older in Ghana is projected to almost double by 2050 (3). Despite their growing numbers, the health and care of the older adult population has not been accorded much attention in Ghana or many other low- and middle-income countries. This has implications for health care provision, as well as social and economic security issues for the older population (4).

This study is based on the World Health Organization's (WHO) multicountry Study on global AGEing and adult health (SAGE) Wave 1 from Ghana, whose findings on household characteristics of the population have been described previously in Biritwum et al. (5). The aim of the current paper is to describe the burden of chronic health conditions and prevalence of contributing risk factors of older adults in contemporary Ghana. A more detailed investigation of hypertension (both self-reported and measured) and arthritis, the two most prevalent health conditions, is also presented. This overall objective is to generate results that help drive the issue of chronic diseases onto the agenda of the Ministry of Health/Ghana Health Service and push forward the implementation of the National Ageing Policy by the Ministry of Employment and Social Welfare.

## Materials and methods

### The setting

Ghana has a population of over 24.6 million people, with 12% aged 50-plus years and 6.5% aged 60-plus years (3). The per capita income is approximately US$670, and they have access to public and private health services through the National Health Insurance Scheme (NHIS) (6). The 2007/08 SAGE Ghana Wave 1^7^ builds on the 2003/04 World Health Survey (WHS), also referred to as SAGE Wave 0 (8). The SAGE Wave 1 round of data collection included follow-up respondents taken from the Wave 0, and added new respondents to increase the cohort size for future waves.

### Sampling design

Ghana used a stratified, multistage cluster design. The sample was stratified by administrative region (Ashanti, Brong Ahafo, Central, Eastern, Greater Accra, Northern, Upper East, Upper West, Volta, and Western) and type of locality (urban/rural), resulting in 20 strata, and is nationally representative. Ghana is divided into 10 regions of different ethnicity, socioeconomic levels, and urban–rural composition. Ghana has two distinct socioeconomic zones; the northern regions (Upper East, Upper West, and Northern) are relatively poor compared to the regions in the middle and southern belts. The Greater Accra (the capital region) and the Ashanti (the most populous region) are the most urbanized with higher socioeconomic levels. A total of 10–15 Enumeration Areas (EA) were selected from the strata according to size. Household listings were done for each selected EA. Twenty households with persons aged 50 years and four households with persons aged 18–49 years were then selected for interview.

All persons aged 50-plus in ‘older’ households (households with at least one person aged 50-plus years) were invited to participate, whereas only one person was randomly selected in ‘younger’ households (households with no person aged 50-plus years) for the individual interview. If a selected individual was found to be incapable of completing an interview for reasons of health or cognition, a proxy questionnaire was completed (8). Standardized training in all aspects of the interview was provided to all interviewers. The questionnaires were translated into respective local languages, following a translation protocol, and modified to take into account the local context where needed.

### Variables

Age, sex, marital status, education, place of residency, measures of wealth (used to generate income quintiles), religion, ethnicity, mother tongue, and work history were collected.

### Income quintiles

Wealth or income quintiles were derived from the household ownership of durable goods, dwelling characteristics (type of floors, walls, and cooking stove), and access to services (improved water, sanitation, and cooking fuel) for a total of 21 assets. A two-step random effect probit model was used to generate the quintiles. An asset ladder was first generated based on the endorsement rate of the different assets. This ladder was then used to arrange household on the same scale, based on their asset ownership. The result is a continuous income score, from which quintiles are created (9).

### Assessment of chronic conditions

For all the chronic disease conditions, prevalence estimates were based on self-report by respondents through the question, ‘Has a health care professional ever told you, you have …?’. The rates for four chronic disease conditions (arthritis, angina, asthma, and depression) were also estimated through symptom-reporting and derived algorithms; however, for this paper only self-reported results are presented.

### Hypertension by BP measurement

In addition to self-reported hypertension, BP was also measured in all respondents by trained interviewers using a Boso Medistar Wrist BP Monitor Model S. Respondents were asked to remain seated and relaxed, and the importance of positioning their arm at the level of their heart was emphasized. BP was measured three times, with 1 minute between each measurement. Hypertension was considered to be present if the mean of the last two measurements was ≥140 mmHg (systolic BP) or ≥90 mmHg (diastolic BP), or if the respondent was currently taking anti-hypertensive medications. Normotensive was defined as ≤140 and ≤90 mmHg, and the respondent had not been taking anti-hypertensive medications during the last 12 months. We furthermore classified the hypertensive respondents as follows using the criteria provide by the 7th Joint National Committee of High BP (JNC7) (10):

- Hypertensive not on treatment: Hypertensive at measurement and no medication during the last 2 weeks
- Normotensive on treatment: Normotensive at measurement and on medication during the last 2 weeks
- Hypertensive on treatment: Hypertensive at measurement and on medication during the last 2 weeks.

### Risk factors

Risk factor questions were based on the WHO STEPwise approach to NCD risk factor surveillance (11). Respondents were asked about their current use of tobacco products, including use by inhaling, sniffing, and chewing, as well as the duration and quantity of their daily tobacco use, which refers to any tobacco products, but smokeless tobacco was excluded. Individuals were grouped into categories: never smoker, ex-smoker, and current smoker.

Alcohol content and quantity of commercially available and home-brewed beverages were used to define a ‘standard drink’. Questions were asked about individual consumption patterns, including frequency and quantity. Drinkers were grouped into lifetime abstainer/occasional (never consumed alcoholic beverage or did not consumed alcohol in the last 30 days); non-heavy drinker (social drinkers, consumed alcohol in the last 30 days); infrequent heavy drinker (binge drinkers, 1–2 days per week with five or more standard drinks for men and four or more standard drinks for women in the last 7 days); and frequent heavy drinker (3 or more days per week with five or more standard drinks for men and four or more standard drinks for women in last 7 days).

Diet questions focus on the quantity of fruit and vegetables consumed in a typical 24-hour period. The intent is on food availability and access, as well as known health risks from insufficient consumption, based on WHO recommendations and defined as less than five servings (80 g per serving) on a typical day (7, 12).

Questions about the type and level of physical activity that the respondent undertakes were based on the Global Physical Activity Questionnaire (GPAQ) (13). Specifically, GPAQ differentiates between work and leisure, and recreational and sport-related activities, and records the frequency (number of days) and duration (minutes or hours) of each activity undertaken in the preceding 7 days. Activities are categorized into high physical activity [vigorous intensity activity on at least 3 days achieving a minimum of at least 1,500 MET (metabolic equivalent)-minutes per week or 7 or more days of any combination of walking, moderate or vigorous intensity activities achieving a minimum of at least 3,000 MET-minutes per week], moderate physical activity (3 or more days of vigorous intensity activity of at least 20 minutes per day; 5 or more days of moderate-intensity activity or walking of at least 30 minutes per day; or 5 or more days of any combination of walking, moderate or vigorous intensity activities achieving a minimum of at least 600 MET-minutes per week), and low physical activity (a person not meeting any of the above mentioned criteria).

BMI results are based on measured weight and height. BMI was calculated by dividing weight (in kilograms) by height (in meters) squared (kg/m^2^). BMI categories are based on WHO recommendations: underweight≤18.4; normal 18.5–24.9; overweight 25.0–29.9; obese ≥30.0 (14).

A Gulick measuring hoop was used to measure waist and hip circumferences. Waist circumference and WHR are important indicators of overall health risk for cardiovascular and metabolic diseases. High risk was defined as a WHR ratio greater than 0.90 cm for men and 0.85 cm for women (15).

### Statistical analysis

The comparison between categorical variables was performed through the Chi-square test. The logistic regression analysis was performed using the SAS survey procedure, which produces estimates from complex sample survey data. Multivariable stepwise logistic regression models were used to examine the association of a range of characteristics (age, sex, residence, education level, income, BMI, tobacco use, alcohol consumption, physical activity, dummies for regions), with the following outcomes: hypertension on measurement, self-reported hypertension, and arthritis. The hypertension models are adjusted for tobacco use and alcohol consumption. The arthritis model is adjusted for level of physical activity. Goodness of fit was evaluated by plotting the estimated values versus residuals and through the Hosmer and Lemeshow's test; multicollinearity was checked by computing the tolerance and the variance inflation. Significance level was set at 0.05. All analyses are weighted and performed using SAS version 9.2.

### Weights

Data on strata sizes and household sizes for selected EA were obtained and used to calculate weights for individual respondents. Individual weights were generated using selection probabilities at each stage of selection, and were post-stratified by region, locality, sex, and age groups (18–49, 50–59, 60–69, 70+) according to the 2009 projected population estimates provided by Stats Ghana (16).

## Results

### Response rate

The overall response rate was 95.9%, ranging from a low of 88.0% in the Upper East region to a high of 100% in the Central region (Table 1).

**Table 1:** Number of respondents (18–49 and 50-plus year age groups) included in final sample^a^ (follow-up from SAGE Wave 0 and new in SAGE Wave 1) and response rate, by region and residence

	Follow-up sample included from SAGE Wave 0 (*n*)		New sample in SAGE Wave 1 (*n*)		Total sample (*n*)	Response rate (%)
Region	Urban	Rural	Urban	Rural	–	–
Ashanti	117	137	355	238	847	97.9
Brong Ahafo	83	123	139	183	528	98.1
Central	63	106	157	240	566	100.0
Eastern	73	128	162	330	693	93.8
Greater Accra	109	20	435	72	636	99.2
North	55	154	40	248	497	97.2
Upper East	20	152	32	198	402	88.0
Upper West	13	33	44	107	197	88.3
Volta	62	137	126	236	561	89.7
Western	51	145	145	303	644	98.9
Total	646	1,135	1,635	2,155	5,571	95.9

a Unweighted results.

### Characteristics of respondents by region

Table 2 presents the distribution of selected characteristics by region. Overall and by region, the distribution of individual respondents by sex was similar, while there was a higher prevalence of individuals in the 50–64 age group compared to the 65-plus age group. About 41% of respondents lived in urban areas, reaching values as low as 12.3% in the Upper East region and 15.4% in the Upper West region. Respondents in Greater Accra were overwhelmingly urban dwellers (85.9%). About 70% of respondents were Christian and 15.9% were Islamic, although in the North region the latter constituted 70.7%. The traditional religion, primal indigenous, accounted for 8.9% of respondents, mainly located in the Upper East region (68.2%), and about 5% of respondents reported no religion.

**Table 2:** Selected characteristics (%) of respondents aged 50-plus years,^a^ by region

	Overall (*n*=4,724)	Ashanti (*n*=932)	Brong Ahafo (*n*=400)	Central (*n*=424)	Eastern (*n*=583)	Greater Accra (*n*=623)	North (*n*=403)	Upper east (*n*=281)	Upper west (*n*=171)	Volta (*n*=483)	Western (*n*=424)
Sex											
Men	49.7	55.1	50.7	43.2	46.8	50.8	51.6	47.1	46.6	44.5	53.5
Women	50.3	44.9	49.3	56.8	53.2	49.2	48.4	52.9	53.4	55.5	46.5
Age group (years)											
50–64	55.1	51.0	56.3	52.8	55.8	60.5	57.1	53.7	54.7	54.4	57.0
65–74	26.9	27.8	25.6	28.2	25.2	26.1	23.6	30.3	30.3	27.4	26.8
75-plus	18.0	21.2	18.1	19.0	19.0	13.4	19.3	16.0	15.0	18.2	16.2
Marital status											
Never married	1.3	1.0	1.2	1.4	0.8	2.0	1.7	2.2	1.8	1.1	0.8
Currently married	59.8	59.3	60.2	52.1	56.0	57.9	70.1	71.2	70.7	60.2	54.4
Cohabiting	0.8	0.3	0.7	0.9	1.6	0.9	0.1	0	0	0.5	2.2
Separated/Divorced	12.2	14.1	12.7	17.5	15.5	11.9	2.1	2.5	0.7	11.6	19.4
Widowed	25.9	25.3	25.2	28.1	26.1	27.3	26.1	24.0	26.9	26.6	23.2
Education											
No formal education	54.0	48.8	56.1	58.9	41.9	32.2	92.6	88.4	91.6	49.0	46.5
Primary (completed or not)	21.3	22.8	22.8	21.7	32.0	24.0	4.3	8.2	1.4	28.5	19.3
Secondary high school	4.0	3.3	2.8	4.2	3.9	10.2	0	2.9	1.7	4.9	1.6
High school completed	17.1	21.1	15.1	13.0	18.9	27.9	1.7	0.2	1.1	12.8	28.4
College and higher	3.6	4.0	3.2	2.2	3.3	5.6	1.3	0.3	4.2	4.8	4.2
Religion											
None	4.9	5.9	4.0	6.2	5.1	3.8	5.9	0.7	0	5.1	6.4
Christianity	69.6	74.8	78.2	79.4	86.8	84.8	13.9	22.0	39.9	69.0	79.2
Islam	15.9	16.7	15.8	8.6	6.4	7.9	70.7	8.5	20.1	3.8	11.3
Primal indigenous	8.9	1.6	0.8	4.8	0.9	2.2	9.1	68.2	39.0	22.0	2.9
Other^b^	0.7	0	1.0	0	0.4	0.5	0.4	0	0	0.2	0
Mother tongue											
Akan	49.8	80.0	69.6	85.2	62.6	29.9	2.4	0	0	3.2	81.0
English	1.4	2.2	0.8	3.5	0.7	2.1	0.2	0	0	0.3	1.9
Ewe	12.2	2.2	2.9	4.0	5.8	15.8	0.1	0	0.3	81.7	2.8
Ga-Adangbe	8.7	0.3	0.5	1.7	20.6	42.5	0.1	0	0.3	0	1.3
Gruma	0.9	1.0	1.6	0.6	0	0.9	2.7	0.6	0.3	0.2	0.5
Grusi	1.1	1.8	0.5	0	0	0.6	0	8.6	0	0	2.1
Guan	1.3	0.3	2.1	0.6	4.0	0.6	1.3	0	0	3.0	0.1
Mande-Busanga	1.3	0.7	1.9	1.2	0.1	0.2	3.6	6.6	0	0.4	1.8
Mole-Dagbon	3.0	3.5	0.8	0	0.2	1.4	22.8	0	0	0	1.0
Other	20.3	11.0	19.4	3.3	6.1	5.8	66.7	83.8	99.1	11.3	7.4
Ethnicity											
Akan	62.0	88.6	87.5	88.5	60.9	27.7	0	0	0	1.4	88.7
Ewe	9.2	1.6	1.8	2.5	4.2	9.8	0.9	0	37.9	52.4	2.1
Ga-Adangbe	13.3	1.2	3.0	3.3	16.1	30.5	0.9	0	31.0	41.8	2.0
Gruma	7.2	1.6	0.6	1.8	12.7	26.1	10.4	0	0	0.1	1.0
Grusi	1.3	1.4	1.2	0.5	0	1.3	4.3	31.0	31.0	0.2	2.2
Guan	1.7	1.4	2.1	0.8	2.8	0.7	5.0	29.5	0	1.6	0.7
Mande-Busanga	1.9	0.6	1.4	1.3	2.9	1.8	13.4	18.2	0	2.2	0.9
Mole-Dagbon	3.3	3.5	2.4	1.3	0.3	2.1	65.1	21.3	0	0.2	2.5
Residence											
Urban	40.6	47.1	36.3	35.5	34.3	85.9	27.0	12.3	15.4	24.4	37.6
Rural	59.4	52.9	63.7	64.5	65.7	14.1	73.0	87.7	84.6	75.6	62.4
Work history											
Never worked	1.6	2.0	2.5	0	0.1	2.2	0.2	9.3	0.9	0	2.3
Not currently working	29.3	29.0	15.4	27.7	27.7	43.5	27.2	35.8	26.7	28.7	25.6
Currently working	69.1	69.0	82.1	72.3	72.2	54.3	72.6	54.9	72.4	71.3	72.1
Type of employment											
Public	10.5	10.2	6.2	5.6	7.7	23.9	3.6	13.5	8.4	10.2	10.7
Private	3.9	2.8	1.1	2.4	2.5	10.5	4.1	4.5	1.6	2.3	4.7
Self-employed	78.3	73.3	91.7	89.2	75.6	61.2	78.6	79.3	90.0	84.2	82.9
Informal	7.3	13.7	1.0	2.9	14.2	4.0	13.7	2.7	0	3.3	1.7
Income quintile											
Q1 (lowest)	18.4	11.0	15.0	20.1	14.6	5.4	29.4	48.7	36.0	22.4	17.9
Q2	19.4	16.5	15.6	23.6	19.7	10.5	23.6	26.8	29.5	24.6	18.7
Q3	20.7	20.2	25.6	29.4	23.9	11.6	21.8	12.9	22.0	22.3	19.1
Q4	20.0	23.7	25.7	16.5	25.1	20.8	17.3	7.1	4.1	16.8	23.3
Q5 (highest)	21.5	28.4	18.1	10.4	16.7	51.7	7.9	4.5	8.4	13.9	21.0

a Weighted estimates.

b Other religions: Buddhism, Chinese, Hinduism, Judaism, and others.

Almost half of the respondents were from the Akan-speaking group (49.8%) by mother tongue and were of Akan ethnicity (62.0%), with relevant regional differences. The Akan-speaking and -ethnic group represented the majority of respondents in Ashanti, Brong Ahafo, Central, and Eastern regions. With respect to mother tongue, the next most common group was the Ewe respondents (12.2%), with the Ga-Adangbe speakers forming the third largest group (8.7%). The positions of the Ewe and Ga-Adangbe groups were reversed for ethnicity. Respondents who spoke Gruma, Grusi, Mande-Busanga, and Mole-Dagbon, the languages of the three northern regions (Northern, Upper East and Upper West), made up 6.3% of the sample. However, with respect to ethnicity, respondents from these three regions made up 10.6%.

Fifty-four percent of the respondents had no formal education, with the three northern regions having more than 88% of their inhabitants with no formal education, which is also reflected by the highest proportion of people in the lowest income quintile.

### Prevalence of chronic conditions by selected characteristics

Table 3 reports the prevalence of chronic conditions by selected sociodemographic characteristics.

**Table 3:** Prevalence (%) of chronic conditions,^a^ by selected sociodemographic characteristics

	Angina	Arthritis	Asthma	Cataracts	Chronic lung disease	Depression	Diabetes	Edentulism	Self-reported hypertension	Measured hypertension	Stroke
Overall	3.6	13.8	3.3	5.3	0.6	1.9	3.8	3.0	14.2	51.1	2.8
Sex											
Men	2.9	11.5	3.3	5.1	0.6	1.2	3.2	2.6	11.3	51.5	2.7
Women	4.3	16.3	3.4	5.5	0.5	2.5	4.4	3.3	17.4	50.7	2.9
*p*-value	<0.05	<0.0001	ns	ns	ns	<0.001	<0.05	ns	<0.0001	ns	ns
Age group (years)											
50–64	2.6	10.2	2.4	2.9	0.5	1.0	3.8	1.9	12.6	50.0	1.9
65–74	4.7	16.4	4.9	6.9	0.7	2.1	4.2	2.5	17.6	54.2	3.5
75-plus	4.9	20.6	3.8	10.2	0.3	4.1	3.2	6.7	13.7	49.9	4.3
*p*-value	<0.001	<0.0001	<0.001	<0.0001	ns	<0.0001	ns	<0.0001	<0.001	<0.05	<0.001
Marital status											
Never married	0.7	8.7	0.7	6.9	0	0	3.4	0	2.5	31.4	3.9
Currently married	3.0	12.1	2.5	4.8	0.5	1.2	3.9	2.2	12.9	48.0	2.3
Cohabiting	4.9	4.4	5.2	4.5	0	1.8	1.5	0	9.9	35.1	0
Separated/Divorced	2.2	15.6	5.6	5.7	0.9	3.1	2.4	3.7	16.7	57.7	4.5
Widowed	5.6	17.5	4.0	6.3	0.6	2.8	4.3	4.3	16.3	56.5	2.9
*p*-value	<0.001	<0.0001	<0.05	ns	ns	<0.05	ns	<0.05	<0.05	<0.0001	ns
Ethnicity											
Akan	3.5	16.4	4.2	5.5	0.7	2.3	4.4	3.4	15.7	60.2	3.2
Ewe	3.0	8.2	2.9	3.8	0	2.2	2.8	0.9	16.7	51.7	3.0
Ga-Adangbe	5.8	15.1	3.9	7.5	0.3	2.2	5.2	2.6	18.7	57.6	4.7
Gruma	5.7	12.7	2.8	7.0	0	3.0	7.3	1.3	24.5	63.9	2.1
Grusi	0	14.8	4.4	4.5	0	3.0	0	2.2	12.3	57.3	0
Guan	3.7	13.9	1.4	0	1.8	0	6.4	0	10.3	44.1	1.8
Mande-Busanga	2.0	20.1	2.2	8.7	0	1.4	2.9	4.1	18.7	58.7	3.0
Mole-Dagbon	4.5	19.3	2.7	5.6	1.0	0	1.2	4.8	7.0	68.4	5.1
Other	2.5	7.2	1.5	4.2	0.6	0.5	1.2	2.6	6.5	44.5	0.9
*p*-value	ns	<0.0001	<0.05	ns	ns	<0.05	<0.0001	ns	<0.0001	<0.0001	<0.05
Residence											
Urban	3.8	14.0	3.5	6.3	0.8	1.9	6.1	4.0	23.1	59.2	4.3
Rural	3.4	13.7	3.2	4.6	0.3	1.8	2.2	2.2	8.0	45.6	1.7
*p*-value	ns	ns	ns	<0.05	<0.05	ns	<0.0001	<0.001	<0.0001	<0.0001	<0.0001
Income quintile											
Q1 (lowest)	3.0	11.0	2.6	3.3	0.7	0.8	2.2	2.8	5.4	41.6	1.8
Q2	2.9	13.8	3.2	3.8	0.4	2.9	1.8	2.6	7.5	46.8	1.9
Q3	4.2	14.0	3.5	4.9	0.6	2.0	2.5	3.3	10.4	50.6	2.8
Q4	4.0	13.8	4.0	6.1	0.2	2.2	5.0	2.7	18.9	57.2	2.9
Q5 (highest)	3.7	16.1	3.2	8.0	0.7	1.4	6.9	3.3	26.7	57.9	4.2
* p*-value	ns	ns	ns	<0.0001	ns	<0.05	<0.0001	ns	<0.0001	<0.0001	<0.05

a Weighted estimates. ns=not significant

*Angina* was reported by 3.6% of the respondents and was significantly more common in women (4.3%) than men (2.9%) (*p*<0.05). Angina reporting increased with increasing age, from 2.6% in those aged 50–64, reaching 4.9% among the 75-plus age group (*p*<0.001). Widowed respondents had higher levels of angina than the currently married.

*Arthritis* was reported by 13.8% of the respondents, with higher prevalence in women (16.3%) than men (11.5%) (*p*<0.0001). Rates showed higher values with increasing age for arthritis, with a 20.6% with arthritis among the 75-plus age group compared to a 10.2% among the youngest age group (*p*<0.0001). Widowed respondents reported more arthritis (17.5%) than those currently married (12.1%).

*Asthma* prevalence was 3.3%, equally reported by both sexes, but with a higher prevalence among the 65–74 age group (4.9%) (*p*<0.001).

*Cataracts* were reported by 5.3% of the respondents, with significantly higher levels for people aged 75 and over (10.2%) (*p*<0.0001), people living in the rural areas (*p*<0.05) and in the wealthiest quintile (*p*<0.0001).

*Chronic lung diseases* prevalence was 0.6% overall, with statistically higher levels in urban than rural residents (0.8% compared to 0.3%, respectively).

*Depression* was reported by 1.9% of the respondents. It was more common among women (2.5%) than men (1.2%) (*p*<0.001), showed higher rates with increasing age (*p*<0.0001) and the lowest income group had the lowest prevalence rate (0.8%) (*p*<0.05).

*Diabetes* was reported by 3.8% of respondents, with higher prevalence in women (4.4%) than men (3.2%) (*p*<0.05), but no difference by age group. Diabetes rates were statistically higher in urban (6.1%) than rural dwellers (2.2%), and the higher-income quintiles reported more diabetes (Q5=6.9% and Q1=2.2%) (*p*<0.0001).

*Edentulism* showed no difference by sex, but respondents aged 75 years and over have a higher prevalence (6.7%) (*p*<0.0001). Urban residents have a higher prevalence (4.0%) than rural residents (2.2%) (*p*<0.001).

*Self-reported hypertension* was reported by 14.2% of the respondents with a higher prevalence in women than men [17.4% compared to 11.3%, respectively, (*p*<0.0001)]. The prevalence of reported hypertension was the highest for the 65–74 age group (17.6%) (*p*<0.001). Hypertension was more prevalent in urban (23.1%) than rural dwellers (8.0%) (*p*<0.0001). The higher-income quintiles reported more hypertension (Q5=26.7% versus Q1=5.5%) (*p*<0.0001).

As expected, prevalence of *measured hypertension* (51.1%) is higher than self-reported hypertension (14.2%). Among the hypertensive, we found very low prevalence of people whose treatment is effective (normotensive on treatment=4.1%), a higher percentage of people whose treatment is not effective (hypertensive on treatment=13.3%) and 82.6% are not on treatment (data not shown).

*Stroke* was reported by 2.8% of respondents. It increased with age (*p*<0.001) and was reported more by urban (4.3%) than rural dwellers (1.7%) (*p*<0.001). The highest income quintile group reported more stroke than the lowest income group (Q5=4.2% and Q1=1.8%) (*p*<0.05).

### Health behaviors by selected characteristics

Table 4 presents the prevalence of health behaviors by selected sociodemographic characteristics.

**Table 4:** Health behaviors (% and 95% CI),^a^ by selected demographic characteristics

Characteristics	Current smoker (daily/not daily)	Infrequent/frequent heavy drinkers	Insufficient intake of fruit and vegetables	Low level of physical activity	Obesity	High-risk WHR
Overall	8.1 (7.0–9.2)	2.0 (1.5–2.5)	67.9 (65.2–70.5)	25.7 (23.1–28.3)	9.7 (8.3–11.1)	77.6 (76.0–79.2)
Age group (years)						
50–64	8.3	2.7	66.4	19.2	12.1	75.3
65–74	8.5	1.2	69.2	29.6	7.1	79.4
75-plus	7.0	1.2	70.4	39.4	6.4	82.0
*p*-value	ns	<0.0001	ns	<0.0001	<0.0001	<0.001
Sex						
Men	13.0	3.1	68.9	21.8	6.3	67.0
Women	2.8	0.8	66.7	29.9	13.6	89.4
*p*-value	<0.0001	<0.0001	ns	<0.0001	<0.0001	<0.0001
Residence						
Urban	5.3	1.8	65.8	37.9	17.6	78.2
Rural	10.1	2.1	69.3	17.2	4.3	77.2
*p*-value	<0.0001	<0.0001	<0.05	<0.0001	<0.0001	ns
Education						
No formal education	8.2	0.7	71.6	25.5	6.8	81.0
Primary (completed or not)	8.3	2.7	65.1	25.2	10.5	76.8
Secondary high school	11.5	3.8	72.5	30.0	21.9	72.3
High school completed	8.1	4.6	60.1	23.7	13.4	70.6
College and higher	2.4	2.4	61.8	37.0	16.9	75.3
*p*-value	<0.0001	<0.0001	<0.0001	<0.0001	<0.0001	<0.0001
Marital status						
Never married	14.0	1.5	65.9	27.4	6.9	70.9
Currently married	9.8	2.3	67.7	21.5	8.3	71.8
Cohabiting	11.3	0	82.1	34.3	3.0	59.8
Separated/Divorced	7.5	2.8	68.3	28.0	13.4	83.5
Widowed	4.3	1.1	68.0	33.0	11.5	88.6
*p*-value	<0.0001	<0.0001	ns	<0.0001	<0.0001	<0.0001
Income quintile						
Q1 (lowest)	14.3	2.3	74.4	16.9	2.7	75.6
Q2	9.6	1.0	69.6	21.0	4.0	78.4
Q3	7.6	2.5	68.3	21.1	7.0	77.7
Q4	6.3	2.7	66.6	31.5	10.7	77.4
Q5 (highest)	4.2	1.5	61.8	36.1	22.3	78.6
*p*-value	<0.0001	<0.0001	<0.0001	<0.0001	<0.0001	ns

Note: Current smoker=daily or non-daily consumption of tobacco in any form; infrequent heavy drinker=1–2 days per week with 5-plus standard drinks for men and 4-plus for women in last 7 days; and frequent heavy drinker=3-plus days per week with 5-plus standard drinks for men and 4-plus for women in last 7 days; insufficient fruit and vegetable intake=<5 servings/day; low physical activity=not meeting criteria for moderate or vigorous activity over the previous week; obesity=BMI>30; high-risk WHR=≥1.0 for men and ≥0.85 for women.

a Weighted estimates.

### Tobacco use

The prevalence of current smokers was 8.1% and was higher among men, rural respondents, those with secondary education, the never married, and those in the lowest income quintile.

### Alcohol consumption

About 0.9% of respondents were infrequent heavy drinkers and 1.1% frequent heavy drinkers. The proportion of infrequent/frequent heavy drinkers was higher for younger respondents, among men, rural respondents, respondents with high school completed, the separated/divorced, and respondents in the fourth income quintile.

### Intake of fruit or vegetables

About 68% of respondents had insufficient daily intake of fruit/vegetables. Over two-thirds of both men and women had insufficient intake of fruit and vegetables in their diet, with no distinction between the sexes. Insufficiency of fruit and vegetable intake worsened marginally with increasing age and among the rural population. There was a clear gradient of dietary intake of fruit and vegetables with the household income quintiles. With regard to educational levels no clear association was found, while the cohabiting respondents generally had a higher prevalence of insufficient intake of fruit and vegetables.

### Physical activity

About 26% of the respondents had engaged in low levels of physical activity. Older respondents, women, urban respondents, respondents with college or higher, currently married, and respondents in the highest income quintile were those with higher prevalence of low levels of physical activity.

### Obesity and WHR

Just 9.7% of the respondents are obese by BMI categories, and 77.6% have a high-risk WHRs. Obesity had higher prevalence among younger age group, female, respondents with high school completed, separated/divorced, and respondents in the highest income quintile.

### Comparison of predictors between self-report and measured hypertension and arthritis

Table 5 shows the ORs for measured and self-reported hypertension, adjusted for tobacco use and alcohol consumption. As a whole, only residence and the BMI appear to be associated with both hypertension outcomes (rural residence and underweight are protective factors, while overweight and obesity are risk factors) and with similar statistical measure of strength. Not significantly associated with the type of hypertension are primary and secondary education, and living in the Eastern and Volta regions.

**Table 5:** Adjusted odds ratios (aORs) (95% CI) for the two highest prevalence conditions, measured/self-reported hypertension and arthritis, by selected sociodemographic characteristics

	Measured hypertension^a^	Self-reported hypertension^a^	Arthritis^b^
Sex			
Men	1	1	1
Women	1.14 (0.94–1.38)	1.47 (1.12–1.93)	1.54 (1.25–1.91)
Age group (years)			
50–64	1	1	1
65–74	1.28 (1.05–1.55)	2.03 (1.55–2.64)	1.90 (1.50–2.41)
75-plus	1.14 (0.90–1.45)	1.63 (1.21–2.19)	2.42 (1.84–3.19)
Residence			
Urban	1	1	–
Rural	0.77 (0.61–0.97)	0.53 (0.39–0.72)	–
BMI			
Normal	1	1	1
Underweight	0.63 (0.50–0.80)	0.66 (0.43–1.00)	1.22 (0.88–1.68)
Overweight	1.72 (1.40–2.11)	1.86 (1.46–2.37)	1.29 (0.97–1.72)
Obese	2.03 (1.53–2.71)	2.40 (1.70–3.38)	1.65 (1.14–2.39)
Education			
No formal education	1	1	–
Primary (completed or not)	1.07 (0.89–1.30)	1.28 (0.92–1.79)	–
Secondary high school	0.90 (0.60–1.35)	1.67 (0.90–3.10)	–
High school completed	1.15 (0.90–1.46)	2.07 (1.46–2.92)	–
College and higher	0.67 (0.43–1.06)	1.97 (1.15–3.34)	–
Region			
Greater Accra	1	1	1
Ashanti	1.14 (0.78–1.66)	0.62 (0.40–0.95)	0.93 (0.57–1.52)
Brong Ahafo	0.98 (0.66–1.45)	0.57 (0.34–0.97)	0.98 (0.53–1.85)
Central	1.03 (0.69–1.52)	0.40 (0.26–0.62)	0.56 (0.29–1.08)
Eastern	1.03 (0.73–1.45)	0.68 (0.44–1.05)	0.87 (0.48–1.58)
North	0.90 (0.54–1.49)	0.20 (0.10–0.42)	0.65 (0.30–1.43)
Upper East	0.37 (0.24–0.58)	0.14 (0.02–1.07)	0.20 (0.09–0.43)
Upper West	0.24 (0.12–0.46)	0.35 (0.09–1.33)	0.03 (0.01–0.25)
Volta	0.99 (0.65–1.48)	0.76 (0.46–1.28)	0.60 (0.33–1.08)
Western	0.81 (0.54–1.21)	0.48 (0.33–0.71)	0.93 (0.56–1.54)

a Models are adjusted for tobacco use and alcohol consumption.

b Model is adjusted by level of physical activity. Residence and education are not significant for arthritis.

All other predictors behave differently: women, people aged 75 and over, people with at least high school education are more likely to self-report hypertension, while no associations are found with measured hypertension. With regards to the regions, people living in Ashanti, Brong Ahafo, Central, North, and Western are less likely to self-report hypertension, whilst people living in Upper East or Upper West are less likely to have hypertension.

Table 5 also presents the ORs for arthritis, the second most commonly reported condition. Being female, increasing age, and obesity carry an increased risk of reporting arthritis. Among the regions, only residents in Upper East or Upper West are less likely to report arthritis with respect to residents in Greater Accra.

## Discussion

This study provides recent prevalence estimates for a range of chronic diseases and underlying risk factors in a nationally representative sample of adult population of Ghana. This study has improved on the approach of the previous nationwide study of the World Health Study/Study on global AGEing and adult health Wave 0 (17) by including, for example, BP and anthropometric measurements. Rates of arthritis, cataracts, and hypertension all exceeded 5% in SAGE Ghana Wave 1, with angina, asthma, diabetes, and edentulism all between 3 and 4%. Only stroke (2.8%), depression (1.8%), and chronic lung disease (0.6%) had prevalence less than 3%.

The under- or over-estimation of chronic disease/risk through self-report is of great concern for epidemiological studies (18). For this study, underestimation was evident in the case of self-reported hypertension. The prevalence of self-reported hypertension was 14.2%, while the prevalence of measured systolic and/or diastolic hypertension was 51.1% among the older population of Ghana. This analysis showed that among the hypertensive, the percentage of persons whose treatment is effective was very low (normotensive on treatment=4.1%), and a relatively higher percentage of persons whose treatment is not effective (hypertensive on treatment=13.3%). Most older adults who are hypertensive using the JNC7 criteria are not on treatment (82.6%).

The prevalence of hypertension in two African sites [South Africa (57.1%) and Zimbabwe (55.6%)] participating in PURE, and using the same self-report and measured BP criteria as this study, was slightly higher than in Ghana (19). Some community surveys in Ghana showed similarly high prevalence; for example, a community study in Greater Accra on adults (aged over 20 years) had hypertension prevalence of 28% and another in women (23.7%) (20, 21). In the same surveys, treatment levels for hypertension were found to be low. This is possibly due to patronage of alternative or traditional systems of care common throughout Ghana, and may point to limitations of this study to capture these treatments. Unfortunately, the findings on treatment levels in Ghana are consistent with results in Senegal and South Africa (22, 23). Addo and colleagues, in a review of population-based hypertension surveys in Ghana from 1973 to 2009, showed that the prevalence of hypertension was higher in urban than rural areas and increased with increasing age with prevalence ranging from 19.3% in rural to 54.6% in urban locations (24). Another systematic review on hypertension in Ghana showed the prevalence to range from 19 to 48% between studies and the prevalence was higher in urban than rural locations (25). The finding on rural-urban differences in the prevalence of hypertension in the SAGE Wave 1 analysis conforms to that found in a rural-urban assessment of hypertension in the Ashanti region of Ghana where the age-adjusted mean systolic and diastolic BP levels were lower in rural men and women than in urban men and women (26).

Socioeconomic development is often accompanied by lower physical activity levels and more sedentary lifestyles. The nature of the common occupations in cities also increases sedentary lifestyles. Older adults in the poorer regions (Northern, Upper West, and Upper East) may walk several kilometers to their farms to engage in physically taxing farming activities. However, the study also found that the Upper East and Upper West regions have the highest prevalence of current smokers, non-heavy drinkers, and those with insufficient intake of fruits and vegetables: major risk factors for the development of hypertension and complications of hypertension (stroke, angina, and other cardiovascular conditions) (27).

Higher-income and urban residence may increase sedentary lifestyles and dietary modification as risk factors for hypertension (24, 25, 27). Stress-related, sedentary, and affluent lifestyle related conditions such as hypertension and diabetes were linked to the urban, high-income older adults in Ghana: the prevalence of stroke, diabetes, and hypertension were clearly higher among urban residents. This is also supported by the assessment of health behaviors and risk for NCDs among older persons in SAGE Wave 1, which indicate higher prevalence of obesity and high-risk WHR among urban residents and higher-income groups compared to rural residents and lower-income groups. In addition, urban residents had higher prevalence of low level physical activity compared to rural residents.

While arthritis, presumably as a result of a lifetime of manual labor would be more prevalent among rural, lower-income older persons, as most rural Ghanaians are engaged in farming as the major occupation (3). It would have been consistent with a higher prevalence of arthritis among older rural persons; however, in this study the prevalence was highest in urban locations and in highest income groups. The regional distribution and location (rural or urban) of both the prevalence and the risk factors are of particular interest which should drive the policy makers intervene to reduce the regionally relevant high burden of chronic conditions among older populations.

Living arrangements and marital status have implications for the risk of poverty in later life, but in different ways for men and women (28). Respondents who were widowed had high prevalence of all the conditions studied, including edentulism and cataracts compared to the prevalence in their currently married counterparts.

The extent to which the rise in prevalence of NCDs is due to a nutritional mismatch in fetal and adult life is unknown; however, dietary interventions at specific time points in the life course may be important for reducing disease risk (29). Education about the importance of adequate intake of fruits and vegetables in the diet for all age groups of the Ghanaian population is critical. The intake of fruits and vegetables was inadequate among the different sociodemographic groups and across regions. The national aging policy needs, as a matter of necessity, to include nutrition among older adults as a critical component if the nation is to preserve and protect the lives of the increasing older population.

Investing in health care and rehabilitation for older persons extends their healthy and active years, a worthy investment by the national government (4). The total expenditure on health in Ghana as a percentage of GDP is 6% and a total government expenditure on health as a percentage of total government spending of 5.5% (8). Private expenditure on health as a percentage of total spending on health is quite high (64%), of which 78.9% of the private spending on health is out-of-pocket expenditure (6).

In our setting, other risk reduction measures such as improvement in access to health and social services might engender more health and social benefits considered in national policies and programs. The wellbeing of older people is not only affected by ‘higher profile’ chronic conditions, such as cardiovascular disease, diabetes, and arthritis, but also by such conditions as edentulism and visual impairment (mainly due to cataracts) as demonstrated in this study. These are health conditions that are easily preventable and should not be disabling older Ghanaians. Access to health care by the aged and supportive social services is essential if Ghana is to improve the quality of life of older persons in society. The management of these chronic conditions is covered under the NHIS (30), with the possibility of using longitudinal data from SAGE Ghana Wave 2 to assess the impact on Ghanaians under the NHIS.

This study had several limitations. Firstly, the self-report of health conditions, such as angina, depression, and hypertension, is likely an underestimate of prevalence rates (31, 32). Methods to improve estimates were employed in SAGE but not presented here (17). The results presented here are based on cross-sectional data from Wave 1, and do not take into account longitudinal data available from Wave 0 of SAGE Ghana, so therefore do not ascribe causality to any of the associated factors in this paper.
